# Production of Sm-153 With Very High Specific Activity for Targeted Radionuclide Therapy

**DOI:** 10.3389/fmed.2021.675221

**Published:** 2021-07-19

**Authors:** Michiel Van de Voorde, Charlotte Duchemin, Reinhard Heinke, Laura Lambert, Eric Chevallay, Thomas Schneider, Miranda Van Stenis, Thomas Elias Cocolios, Thomas Cardinaels, Bernard Ponsard, Maarten Ooms, Thierry Stora, Andrew R. Burgoyne

**Affiliations:** ^1^Belgian Nuclear Research Center, Institute for Nuclear Materials Science, Mol, Belgium; ^2^Department of Physics and Astronomy, Institute for Nuclear and Radiation Physics, KU Leuven, Leuven, Belgium; ^3^European Organization for Nuclear Research, MEDICIS, Geneva, Switzerland; ^4^European Organization for Nuclear Research, Thin Film Lab, Geneva, Switzerland; ^5^Department of Chemistry, KU Leuven, Leuven, Belgium

**Keywords:** radiolanthanides, samarium-153, theranostics, targeted radionuclide therapy, cancer, nuclear medicine, mass separated radionuclides, resonant laser ionization

## Abstract

Samarium-153 (^153^Sm) is a highly interesting radionuclide within the field of targeted radionuclide therapy because of its favorable decay characteristics. ^153^Sm has a half-life of 1.93 d and decays into a stable daughter nuclide (^153^Eu) whereupon β^−^ particles [E = 705 keV (30%), 635 keV (50%)] are emitted which are suitable for therapy. ^153^Sm also emits γ photons [103 keV (28%)] allowing for SPECT imaging, which is of value in theranostics. However, the full potential of ^153^Sm in nuclear medicine is currently not being exploited because of the radionuclide's limited specific activity due to its carrier added production route. In this work a new production method was developed to produce ^153^Sm with higher specific activity, allowing for its potential use in targeted radionuclide therapy. ^153^Sm was efficiently produced via neutron irradiation of a highly enriched ^152^Sm target (98.7% enriched, σ_th_ = 206 b) in the BR2 reactor at SCK CEN. Irradiated target materials were shipped to CERN-MEDICIS, where ^153^Sm was isolated from the ^152^Sm target via mass separation (MS) in combination with laser resonance enhanced ionization to drastically increase the specific activity. The specific activity obtained was 1.87 TBq/mg (≈ 265 times higher after the end of irradiation in BR2 + cooling). An overall mass separation efficiency of 4.5% was reached on average for all mass separations. Further radiochemical purification steps were developed at SCK CEN to recover the ^153^Sm from the MS target to yield a solution ready for radiolabeling. Each step of the radiochemical process was fully analyzed and characterized for further optimization resulting in a high efficiency (overall recovery: 84%). The obtained high specific activity (HSA) ^153^Sm was then used in radiolabeling experiments with different concentrations of 4-isothiocyanatobenzyl-1,4,7,10-tetraazacyclododecane tetraacetic acid (*p*-SCN-Bn-DOTA). Even at low concentrations of *p*-SCN-Bn-DOTA, radiolabeling of 0.5 MBq of HSA ^153^Sm was found to be efficient. In this proof-of-concept study, we demonstrated the potential to combine neutron irradiation with mass separation to supply high specific activity ^153^Sm. Using this process, ^153^SmCl_3_ suitable for radiolabeling, was produced with a very high specific activity allowing application of ^153^Sm in targeted radionuclide therapy. Further studies to incorporate ^153^Sm in radiopharmaceuticals for targeted radionuclide therapy are ongoing.

## Introduction

Targeted radionuclide therapy (TRNT) has proven to be successful in oncology over the last decade (1–5). In TRNT a radionuclide is linked to a molecule that selectively binds to over-expressed receptors of cancer cells, allowing for a targeted approach in cancer therapy. Accumulation of the radiopharmaceutical in tumor tissue delivers toxic levels of radiation directly to the malicious tumor cells minimizing dose given to healthy tissue.

The use of rare earth elements (REEs) in TRNT have been especially investigated intensively because of their favorable decay characteristics for nuclear medicine applications (6–14). The application of several drugs, containing REE radionuclides (^90^Y, ^153^Sm and ^177^Lu), have been approved in nuclear medicine, while the use of others are being studied (^149, 152, 155, 161^Tb, ^166^Ho, ^169^Er, ^167^Tm, and ^175^Yb). Fortunately many of the therapeutic REE radionuclides can be easily produced in a nuclear research reactor with high yields and high specific activities.

^153^Sm has a half-life of 1.93 d and decays into a stable daughter nuclide (^153^Eu). Upon decay β^−^ particles (E_max_ = 705 keV, 635 keV) and γ photons (103 keV) are emitted which are suitable for therapy and SPECT imaging, respectively. Therefore, ^153^Sm is a very interesting radioistope in theranostic clinical applications of nuclear medicine. ^152^Sm has a high thermal neutron cross section (σ_th_ = 206 barn), which allows for efficient neutron activation of ^152^Sm via an n,γ reaction to produce ^153^Sm [^152^Sm (n,γ) ^153^Sm] in a nuclear research reactor with a high thermal neutron flux using highly enriched ^152^Sm target material. Despite its highly interesting decay characteristics for nuclear medicine, the use of ^153^SmCl_3_ (as Quadramet) is currently restricted to bone pain palliation, for patients suffering bone metastases originating from various types of cancer. This is a consequence of the direct carrier added production route. Considering a 2 ×10^14^ neutrons/cm^2^/s thermal neutron flux, at the end of irradiation (EOI) a maximum specific activity of ≈135 GBq/mg can be reached after 13 days of irradiation. Neglecting the high burn-up of the target, this corresponds to a ratio of 120:1 between ^152^Sm and ^153^Sm nuclides. The specific activity drops exponentially as a function of time after EOI as a result of the fast nuclear decay of ^153^Sm, drastically increasing the ^152^Sm-to-^153^Sm ratio. Chemical isolation of ^153^Sm from its ^152^Sm target matrix is impossible. Therefore, the achievable specific activity of c.a. ^153^Sm remains too low to be used in TRNT. The high content of stable ^152^Sm would make chelation at the needed low ligand concentrations inefficient. Moreover, the excessive amount of ^152^Sm-complex would saturate the receptor sites at the targeted cancer cells with inactive material, leading to a low therapeutic efficiency thus making carrier added ^153^Sm ineffective in TRNT.

^152^Sm targets are typically irradiated for several days to reach high production yields for ^153^Sm. As a consequence of the relatively short half-life of ^153^Sm, trace amounts of the long-lived ^154^Eu (t_1/2_ = 8.6 y) are co-produced by neutron irradiation of the stable ^153^Eu (σ_th_ = 300 b) daughter nuclide. The presence of these long-lived impurities limits the shelf-life of the radiopharmaceutical significantly as they might deliver unacceptable dose to the patient on the long term. Therefore, the ^154^Eu content allowed in radiopharmaceuticals, such as Quadramet, is strictly regulated by international agencies (15, 16).

The use of mass separation on irradiated ^152^Sm targets allows for reaching much higher levels of specific activity of ^153^Sm, i.e. pure non-carrier-added ^153^Sm corresponds to a theoretical specific activity of 16.4 TBq/mg. Moreover, any long-lived ^152^Eu and ^154^Eu impurities are removed from ^153^Sm simultaneously by applying mass separation. The possibility to produce high specific activity (HSA) ^153^Sm will make this radionuclide and its beneficial β^−^ and γ emissions eligible for receptor-targeted β^−^ therapy, leading to the development of various novel radiopharmaceuticals.

In this study, we demonstrated the possibility to perform neutron activation of 98.7% highly enriched ^152^Sm as Sm (NO_3_)_3_ targets using thermal neutron fluxes of 2.0–2.5 ×10^14^ neutrons/cm^2^/s in the BR2 reactor, and to isolate ^153^Sm from its neutron activated ^152^Sm target matrix by means of mass separation at the CERN-MEDICIS facility (17). Mass separation, both on-line and off-line, have proven to be of added value to produce non-conventional radionuclides for medical research (18). Examples include the production of ^149^Tb, ^152^Tb, ^155^Tb, ^169^Er, ^167^Tm, and ^175^Yb (19–21). The MS collection foil was processed in the NURA radiochemistry labs at SCK CEN to obtain ^153^SmCl_3_ with high specific activity (1.87 TBq/mg) and high radionuclidic and chemical purity suitable for TRNT. Radiolabeling experiments using the HSA ^153^Sm demonstrated high radiolabeling yields. This proof-of-concept study confirms the ability to produce ^153^Sm with very high specific activity suitable for radiolabeling and opens perspectives for future large supply.

## Materials and Methods

### Neutron Activation of ^152^Sm

**Figure 1:**
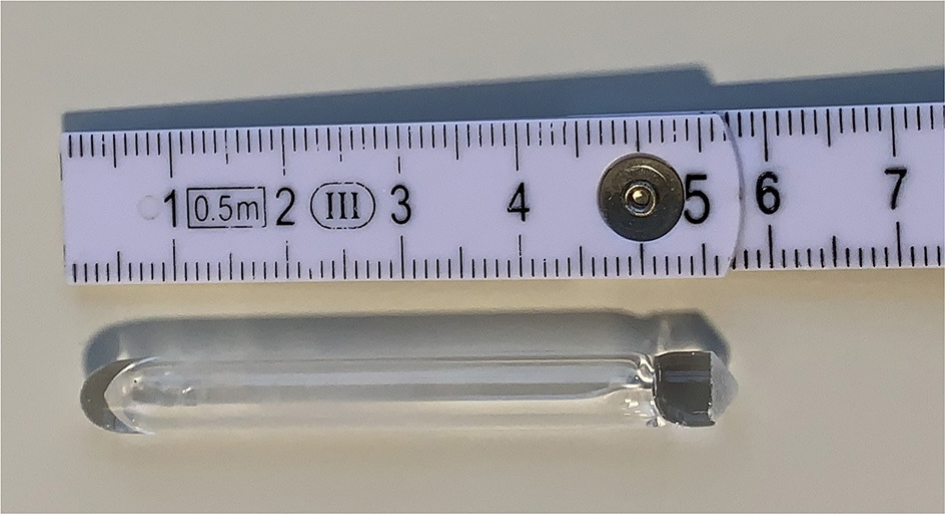
Typical example of a vacuum-sealed quartz ampoule ready for irradiation. The target material is deposited at the bottom of the ampoule (left side here).

Highly enriched ^152^Sm_2_O_3_ (98.7% ± 0.1, Neonest AB) target material was converted to ^152^Sm (NO_3_)_3_ prior to neutron activation to facilitate target handling after irradiation. ^152^Sm_2_O_3_ was dissolved in 4 mol/L HNO_3_ solution (trace metal grade, VWR). Afterwards, the solution was evaporated to dryness using a rotavapor (Büchi) and a vacuum oven (Thermo Fisher). The obtained ^152^Sm (NO_3_)_3_ was dissolved in a known volume of trace metal grade H_2_O (VWR), and dispensed in high-purity quartz glass ampoules (Heraeus). A target mass of 150 μg ^152^Sm was used in the first series of irradiations. After optimization of the processing protocols, the target mass was increased to 350 μg ^152^Sm to allow for gradual up-scaling of the ^153^Sm production, while following the ALARA principle. The target solution inside the quartz ampoules was evaporated to dryness inside a vacuum oven (80 °C, 300 mbar). The quartz ampoules were sealed under vacuum using a custom-made sealing station and a propane/oxygen torch. An example of a vacuum-sealed ampoule with the target material deposited at the bottom is presented in Figure 1. The outer surface of the ampoules underwent thorough cleaning to avoid the undesired activation of any contaminants.

The quartz ampoules were irradiated in the BR2 reactor at SCK CEN following the routine production procedure (22). They were loaded into standard cold-welded “CFS” irradiation capsules containing an aluminum insert and helium gas. The capsules were irradiated in thimble (DG – “Doigt de Gant”) irradiation devices during 2 or 3 days in thermal neutron fluxes of 2.0 to 2.5 ×10^14^ neutrons/cm^2^/s and cooled for 5 days to allow the decay of short-lived radionuclides. The irradiation capsules were sent to the BR2 Hot-Cells for decanning to recover the quartz ampoules, which were then shipped to the radiochemical labs at SCK CEN for opening and target processing. The irradiated target material was dissolved in trace metal grade water and transferred into a tantalum sample boat lined with a rhenium foil provided by CERN. A small aliquot of the solution was taken for analysis by gamma spectrometry. The target material was evaporated to dryness, by using a 350 W infra-red lamp, resulting in the deposition of the irradiated target on the rhenium foil before being shipped to CERN-MEDICIS for mass separation. So far, eight batches underwent mass separation. The activity of the shipped batches increased stepwise over time, ranging from 1.9 to 14.8 GBq of ^153^Sm, to allow safe optimization of the different process steps involved.

### Off-Line Mass Separation of ^153^Sm

After each reception, the sample holder containing the radioactive samarium source was loaded into the empty oven of a standard ISOLDE target and ion source unit (23) at CERN-MEDICIS. The target's oven is made of tantalum and is connected to a rhenium surface ion source via a hot transfer line. Dedicated sample holders have been designed and produced by CERN in order to efficiently and safely load the irradiated target material inside the target container. In addition, the sample holder has been designed to ensure the safe transportation of the irradiated materials from the external institutes to CERN. Once the radioactive material is loaded into the target container, the target unit is coupled to the MEDICIS target station in preparation for the collection. The target station includes a coupling flange held at a potential ranging from 30 to 60kV and an extraction electrode placed after an acceleration gap, at a distance ranging from 55 to 80mm, for a surface ion source, from the ion source's exit. An Einzel lens is used to shape the ion beam downstream of the extraction electrode. Two electrostatic XY deflector stages at 5 kV are located between the Einzel lens and the extraction electrode. The targets are heated to very high temperatures, typically up to 2,300 °C depending on the target material and radionuclide considered, to allow for the diffusion and effusion of the isotopes of interest out of the target to the ion source for subsequent ionization. The ions are then accelerated and sent through a mass separator which is a dipole magnet (24). The dipole magnet has been modified to allow its use with the MEDICIS Laser Ion Source for Separator Assembly (MELISSA) laser laboratory (25), resembling the Ti:sapphire laser setup of the ISOLDE Resonance Ionization Laser Ion Source (RILIS) (26). MELISSA has been in service since April and helps to increase the separation efficiency and the selectivity at CERN-MEDICIS. ^153^Sm and its isobaric daughter ^153^Eu were implanted into a metallic zinc layer that was deposited on a gold foil. These implantation foils were prepared by a 99.995% pure metallic zinc granulate being thermally heated in a molybdenum boat under high vacuum and evaporated onto gold substrates. A zinc layer thickness of 500 nm in this Physical Vapor Deposition (PVD) process was attained and confirmed by the use of a build-in INFICON thickness sensor. The surface of the gold substrates underwent crucial surface roughening and ultrasonic cleaning steps prior to the zinc deposition to allow for proper zinc adherence and layer uniformity.

### Radiochemical Processing of the Mass Separation Target

The implanted ^153^Sm and its daughter ^153^Eu were recovered from the gold foil by dissolving the metallic zinc implantation layer. The metallic zinc layer was readily dissolved in a 4 mol/L HNO_3_ solution (trace metal grade, VWR). The gold foil was rinsed twice with 4 mol/L HNO_3_. The activity of the gold foil was measured in a dose calibrator (Veenstra) before and after the dissolution of the metallic zinc layer to estimate the efficiency of activity removal from the gold foil. The collected fractions were loaded onto a PEEK column (ø: 2.1mm, l: 30mm, Bio-Safe, TrisKem International) packed with DGA extraction chromatography resin (branched, 50–100 μm, TrisKem International) using a peristaltic pump (ISMATEC IPC-8) peristaltic pump. Before being used, the DGA column was conditioned with 10 mL of 4 mol/L HNO_3_. ^153^Sm and its ^153^Eu daughter are retained on the DGA resin in acidic conditions, whereas Zn^2+^ is not. The loaded DGA column was washed excessively with 20 mL of 4 mol/L HNO_3_ to rinse the large amount of Zn^2+^ from the column. The wash solutions were checked for activity in the dose calibrator to ensure no ^153^Sm or ^153^Eu were co-eluted during the washing of the loaded DGA column. ^153^Sm and ^153^Eu were readily eluted off the DGA column using water. The eluted activity was monitored by means of a dose calibrator.

Taking logistics and the relatively short half-life of ^153^Sm into account, the ^153^Sm eluent still contains a high amount of the stable ^153^Eu daughter isotope. This lowers the radiochemical purity drastically. Therefore, ^153^Sm and ^153^Eu are separated using a high-pressure ion chromatography system (HPIC, Shimadzu) equipped with a strong cation exchange column (ø: 6mm, l: 50mm, Shodex IC R-621). To ensure retention on the strong cation exchange column, the pH of the ^153^Sm eluent from the DGA column was adjusted with a 1 mol/L NH_4_OH (trace metal grade, VWR) solution to reach a pH range of 2.5–4.5. After injection on the HPIC system, ^153^Sm was separated from ^153^Eu using a 180mmol/L 2-hydroxyisobutyric acid (α-HIBA) solution, which was adjusted with NH_4_OH (trace metal grade) to pH 4.6. The HPIC system was equipped with a radio-detector (Elysia-Raytest, operated via GINA Star V4.8) to allow on-line monitoring of the activity eluting off the strong cation exchange column.

The collected ^153^Sm fractions were combined and loaded onto a PEEK column (ø: 2.1mm, l: 30mm, Bio-Safe, TrisKem International) packed with LN3 extraction chromatography resin (50–100 μm, TrisKem International) to remove α-HIBA from the purified ^153^Sm. The LN3 column was conditioned with a 1 mol/L HNO_3_ (trace metal grade, VWR) solution and washed with copious amounts of water prior to loading of the ^153^Sm (α-HIBA)_3_ solution. After loading, α-HIBA was washed from the column with water while ^153^Sm was retained on the column. ^153^Sm was eluted from the LN3 column in a concentrated fraction using a 50mmol/L HCl solution (trace metal grade, VWR), obtaining a ^153^SmCl_3_ solution ready for radiolabeling. A small aliquot was taken for gamma spectrometry analysis to determine the specific activity, via co-analysis with ICP-MS, and radionuclidic purity of the produced HSA ^153^SmCl_3_. Chemical purity was determined via ICP-MS. Radiochemical purity was determined via radio-TLC, using a 0.1 mol/L sodium citrate solution (pH 5). In total, four of the retrieved mass separation targets were fully dedicated for analysis and evaluation for optimization of the entire radiochemical purification process, collecting valuable data after each process step.

### Radiolabeling of High Specific Activity ^153^Sm

The suitability of the obtained HSA ^153^SmCl_3_ for radiolabeling was demonstrated with various concentrations of 4-isothiocyanatobenzyl-1,4,7,10-tetraazacyclododecane tetraacetic acid (*p*-SCN-Bn-DOTA, Macrocyclics). 0.5 MBq of HSA ^153^SmCl_3_ was added to different concentrations of *p*-SCN-Bn-DOTA (1, 5 and 10 nmol/L) in 0.1 mol/L NaOAc buffer (pH 4.7) with a total reaction volume of 1 mL. The reactions were incubated in a thermomixer (Eppendorf) for 60 min while maintaining a constant temperature of 30, 60 or 90°C. To obtain robust results, test reactions were performed in triplicate for each reaction condition. After incubation, the radiolabeling yield was evaluated using thin-layer chromatography. 5 μL of each reaction was spotted on a glass microfiber chromatography paper strip impregnated with silica gel (iTLC-SG, Agilent Technologies) and set in an acetonitrile: water mixture (70/30) to allow migration of the ^153^Sm-*p*-SCN-Bn-DOTA complex. The TLC papers were cut in half and the activity of the bottom and top parts of the TLC paper were counted for 2 min each using a gamma counter (Perkin Elmer).

## Results and Discussion

### Off-Line Mass Separation of ^153^Sm

Prior to the reception of radioactive samarium, tests with natural stable samarium sources had been performed with an evaporated sample of Sm_2_O_3_ (in 5% HNO_3_) in order to define the laser excitation scheme for the MELISSA ion source and the optimal temperature window for samarium release. These tests allowed the facility to operate with a reproducible extraction and collection protocol, and to reach 8% of separation efficiency already at the first collection of ^153^Sm, exceeding the best operation performances achieved so far (18).

**Figure 2:**
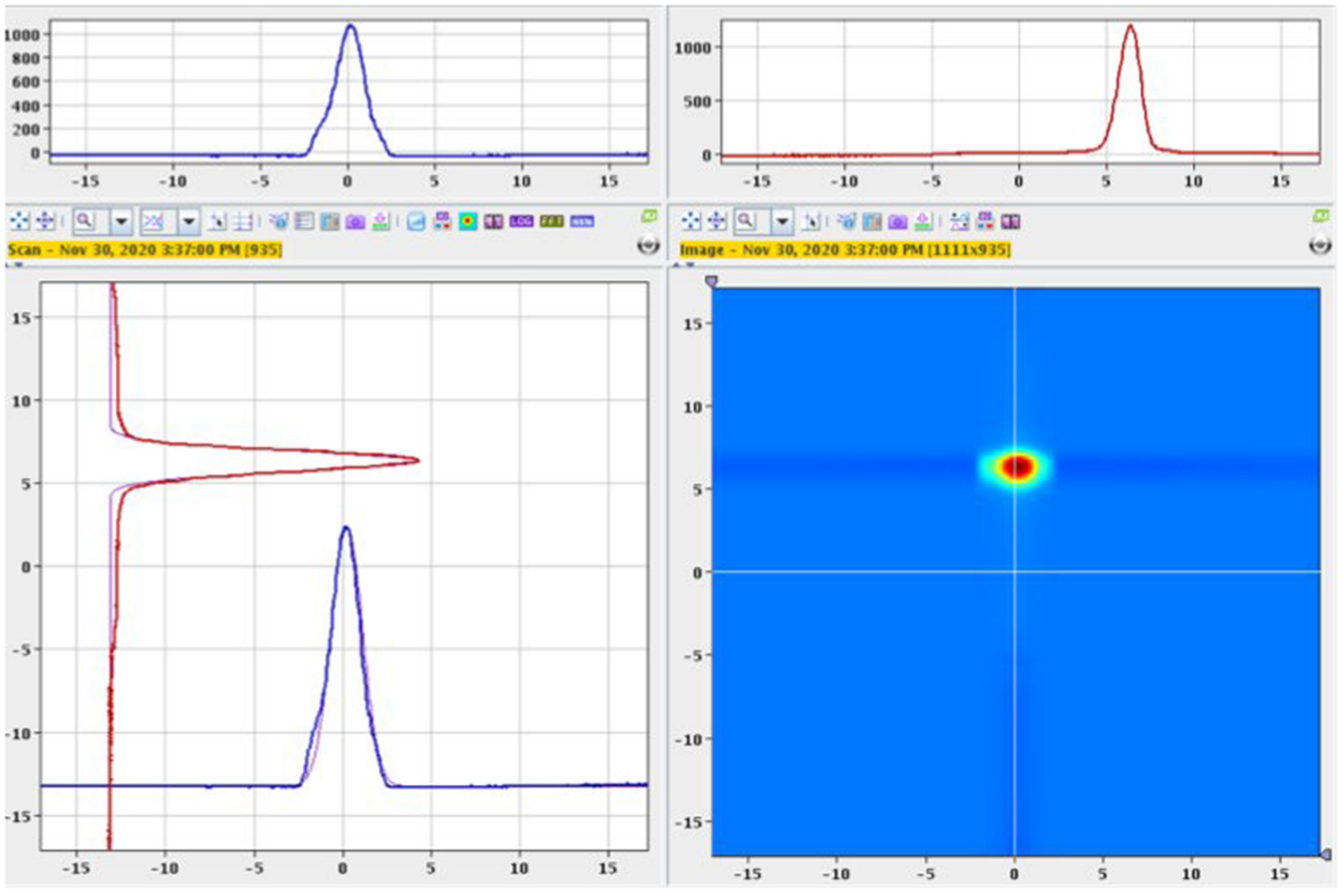
^153^Sm beam profile with σ_horizontal_ = 0.88mm and σ_vertical_ = 0.62mm.

**Figure 3:**
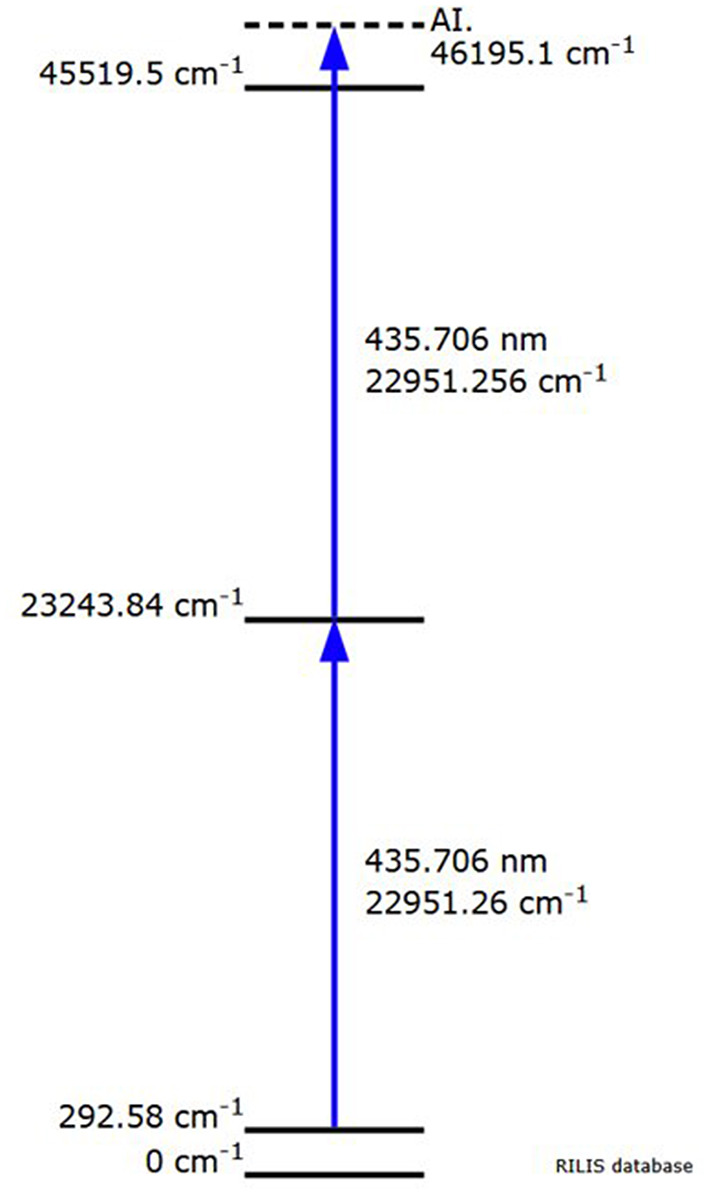
Resonant laser ionization from the low-lying *E* = 292.6 cm^−1^ state of samarium via two subsequent λ_vac_ = 435.706 nm transitions into an auto-ionizing state (RILIS database).

The separator parameters were set with a beam extraction voltage of 60 kV and an acceleration gap of 60mm from the ion source's exit. The target container oven was heated at 1,130 °C by ohmic heating and the ion source line at 2,100 °C for preliminary optimization steps with stable ^152^Sm. Most of the ^153^Sm activity was extracted between 1,640 and 1,900 °C (corresponding to a current of 500–580 A). Figure 2 shows the ion beam profile during implantation for *A* = 153. Samarium was ionized by resonantly exciting the electronic shell from the low-lying *E* = 292.6 cm^−1^ state (17% thermal population at 2,100 °C) via two subsequent λ_vac_ = 435.706 nm transitions into an auto-ionizing state (Figure 3) (27). In the exceptional case that only one laser is required for both transitions enabled alternating pulsing of the two available pump lasers This doubles the standard repetition rate to 20 kHz and increased the laser ionization rate by 30%, compared to synchronous operation at 10 kHz. The ^153^Sm implantation rate in MBq/h was monitored by a compact room temperature gamma-spectrometer (Kromek® GR-1) which was positioned in front of the collection chamber window. The identification and quantification of the implanted ions were based on the observation of the 103.2 keV gamma-line. The average ^153^Sm collection time for the 8 collections performed in 2020 was 36 h with an average efficiency of 4.5% and a maximum separation efficiency of 12.7%. This efficiency was calculated based on the activity measured on the collection foil at the end of the collection as a ratio to the ^153^Sm activity in the target at start of the collection. In 2020, 330 h of MEDICIS operation were devoted to the collection of ^153^Sm. Each collection foil was shipped back to SCK CEN for further radiochemical processing.

### Radiochemical Processing of the Mass Separation Target

The metallic zinc layer, into which the mass-separated ^153^Sm was implanted, was readily dissolved using a mineral acid, oxidizing Zn^0^ to Zn^2+^. By measuring the radioactivity of the gold foil before and after dissolution of the metallic zinc implantation layer, a ^153^Sm recovery of 85–92% was achieved. Activity remaining on the gold foil after the acid washes originates from ^153^Sm that penetrated the metallic zinc layerand got implanted into the gold foil as a result of the high ion beam intensity. This activity could not be removed by the chemical conditions used and was considered a total loss in the production process. No additional efforts were undertaken to dissolve the gold foil, as more aggressive media (e.g., *aqua regia*), would be required.

An extraction chromatography column packed with branched DGA resin (N,N,N′,N′-tetrakis-2-ethylhexyldiglycolamide) was used to remove the large excess of Zn^2+^ from ^153^Sm in highly acidic conditions. DGA resins are known to have large affinities, i.e., large k′ values, for trivalent lanthanides in concentrated HNO_3_ and HCl media (28), following the extraction mechanism Ln^3+^ + 3X^−^ + 3 $\bar{\text{DGA}}$ ⇌ $\bar{\text{Ln}{\text{X}}_{3}{\left(DGA\right)}_{3}}$, with X being NO${}_{3}^{-}$ or Cl^−^. The high affinity of Sm^3+^ for DGA resin allows efficient retention of ^153^Sm on the column. Zn^2+^ ions have much lower affinity for DGA, i.e., a very low k′ value, and are easily eluted off the column. Excessive washing of the loaded DGA column with concentrated HNO_3_ allows for efficient removal of the Zn^2+^ impurities from ^153^Sm. Sm^3+^ can be easily stripped from the DGA column by reducing the nitrate concentration in the mobile phase, i.e., reversing the above extraction mechanism. Quantitative ^153^Sm elution was obtained using water as an eluent. Despite the elution of ^153^Sm from the DGA column in water, the obtained solution remained too acidic for direct loading onto the strong cation exchange column used in the next process step (*vide infra*). The pH of the ^153^Sm fraction was adjusted to 2.5–4.5 using a dilute NH_4_OH solution. Activity measurements of the wash solutions and the collected ^153^Sm fraction resulted in a process efficiency of >99.9%. Chemical purity of the ^153^Sm fraction was found to be highly dependent on the volume wash solution used. 5 mL of 4 mol/L HNO_3_ proved to be insufficient as 60 ppb Zn was still present in solution. Also, some traces of iron (17 ppb), copper (5 ppb) and lead (126 ppb) were found. Therefore, the volume wash solution was increased to 20 mL to further decrease the zinc and lead impurities in the ^153^Sm fraction (Zn <20 ppb, Pb <9 ppb). Small amounts of zinc impurities in the ^153^Sm fraction are, however, not problematic at this stage as they will be further removed in the following process steps. Radionuclidic analysis of this ^153^Sm fraction confirmed that the amount of ^152^Eu, ^154^Eu, and ^155^Eu was below the minimum detectable activity (MDA) limit of the gamma spectrometer. Gamma spectrometry of a non-purified ^152^Sm target irradiated in BR2 at 2 ×10^14^ neutrons/cm^2^/s contained 26 Bq/GBq ^152^Eu, 29 Bq/GBq ^154^Eu and 4 Bq/GBq ^155^Eu at EOI. The absence of these long-lived europium isotopes proves the high separation efficiency and selectivity of the mass separator at CERN-MEDICIS.

**Figure 4:**
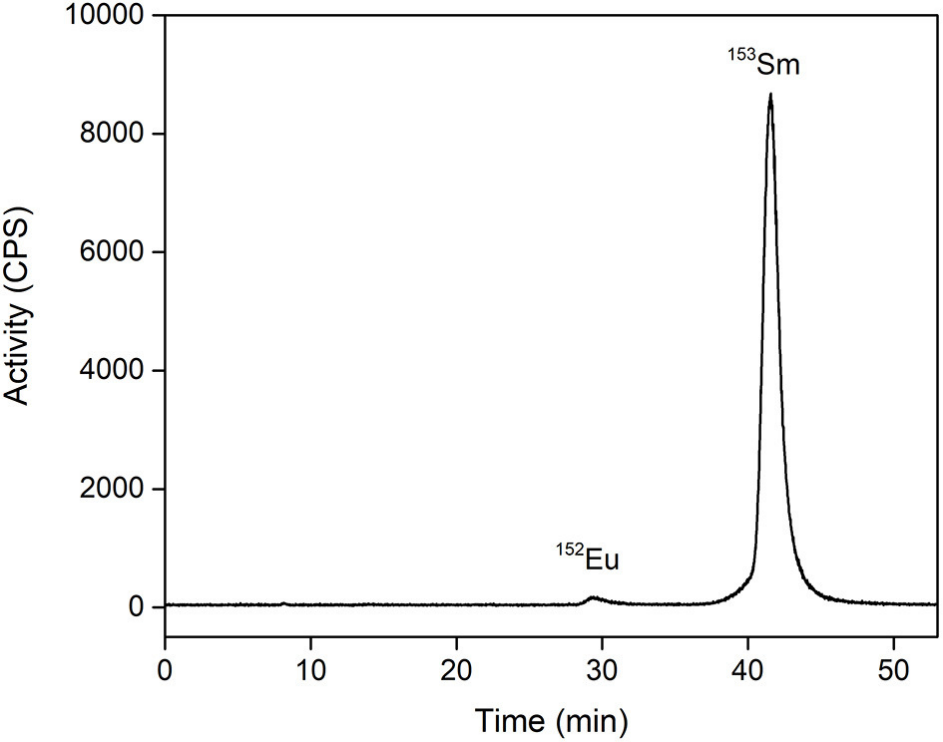
Radio-chromatogram of ^153^Eu/^153^Sm separation on a strong cation exchange column using α-HIBA. Flow rate: 1 mL/min. The feed solution was spiked with ^152^Eu tracers to enable detection of the Eu^3+^ fraction.

The majority of the ions in the ^153^Sm fraction consists of the inactive ^153^Eu daughter isotope. Because samarium and europium possess very similar chemical properties, high amounts of inactive ^153^Eu would significantly decrease the radiolabeling efficiency of ^153^Sm, as well as its use in TRNT. The isobaric ^153^Eu cannot be physically separated from ^153^Sm in a mass separator, and therefore requires a chemical separation step. The use of a resonant laser for selective ionization of Sm prior to mass separation favored collection of ^153^Sm over ^153^Eu but remains less efficient than chemical separation. Moreover, the mass separated ^153^Sm sample had to be transported from Switzerland to Belgium by road. Given the relatively short half-life of ^153^Sm, a significant amount of ^153^Sm decayed into ^153^Eu during transport. Therefore, ^153^Sm was separated from ^153^Eu on a radio-HPIC system using a strong cation exchange column and an aqueous mobile phase comprising the weak acid α-HIBA. The pH of the mobile phase was 4.6, i.e., above the pKa of α-HIBA (4.01). This pH enables the weak acid as well as the functional groups of the stationary phase to interact with the lanthanides. To date, this method proved to be the most efficient in separation of micro amounts of non-carrier-added produced radiolanthanides from macro amounts of redundant target material, being a neighboring lanthanide (13, 29, 30). Sm^3+^/Eu^3+^ separation is a result of the small differences in complex formation between α-HIBA and the lanthanide, and is based on their small difference in ionic radius and charge density as a result of a phenomenon known as lanthanide contraction. A slightly more stable complex is formed between Eu^3+^ and α-HIBA because of its higher charge density. Careful selection of the α-HIBA concentration results in separation of Sm^3+^ and Eu^3+^, with the Eu^3+^ fraction eluting prior to the Sm^3+^ fraction. In the method applied, Sm^3+^/Eu^3+^ separation was achieved using an α-HIBA concentration of 0.180mmol/L at a flow rate of 1 mL/min, with Eu^3+^ eluting from the column after 30 min (Figure 4). The α-HIBA concentration was gradually increased to 0.200mmol/L after elution of the Eu^3+^ fraction to accelerate the elution of the Sm^3+^ fraction. The Sm^3+^ fraction eluted after 42 min. During method development, a ^152^Eu tracer was added to the feed solution to enable on-line detection of the Eu^3+^ fraction with the radio-detector of the HPIC system. A baseline separation with a resolution of 5.22 was observed for Sm^3+^/Eu^3+^. The recovery rate for ^153^Sm after HPIC was determined to be 98–99%.

The ^153^Sm fraction collected after HPIC purification was further processed to remove the α-HIBA, and for up-concentration. This was done by loading the ^153^Sm fraction onto a column packed with LN3 extraction chromatography resin. LN3 resin comprises the organophosphorus extractant di-(2,4,4-trimethylpentyl) phosphinic acid [H (TMPeP)] which is impregnated on an inert polymer support (31, 32). In neutral conditions H (TMPeP) coordinates stronger to trivalent lanthanides than α-HIBA, efficiently extracting the ^153^Sm into the thin organic layer of the LN3 resin via the extraction mechanism: Ln^3+^ + 3 $\bar{\text{H}\left[\text{TMPeP}\right]}$ ⇌ $\bar{\text{Ln}{\left(\text{TMPeP}\right)}_{3}}$ + 3H^+^. α-HIBA is not retained by the LN3 resin and is washed from the column with water. Because of the high k′ value of Sm^3+^ for LN3, ^153^Sm was well-retained on the column in neutral conditions. Sm^3+^ could be efficiently (recovery rate >99.9%) back-extracted to the aqueous mobile phase using a dilute acid solution, i.e., 50mmol/L HCl, as the addition of protons reverses the above extraction mechanism. In the best instance a ^153^SmCl_3_ solution of 67 MBq/mL suitable for radiolabeling was obtained. Analysis of the solution resulted in a specific activity of 1.87 TBq/mg at the time of MS collection, i.e., achieving a ^152^Sm-to-^153^Sm ratio of 8:1. No other radionuclides could be identified above the detection limit of the gamma spectrometer in both the active and decayed sample, resulting in a very high radionuclidic purity. Radiochemical purity of this HSA ^153^SmCl_3_ batch was 98.9 ± 0.24%.

The entire radiochemical process was found to be highly efficient, reaching an average overall recovery rate of 84%. Most of the ^153^Sm is lost at the front-end of the process, as not all activity could be recovered from the MS collection foil. ^153^Sm implanted into the gold foil could not be leached out in the conditions used. The amount of ^153^Sm ending up in the gold foil varied over the different batches, which may indicate a difference in thickness of the metallic zinc layer on the gold foils used.

With the different process steps optimized, longer irradiation times and larger target masses will be used for neutron activation. Higher levels of radioactivity at the front-end of the cycle will automatically result in higher amounts of HSA ^153^Sm that can be used for further pre-clinical evaluation. Higher throughput of radioactivity at the mass separation step will be evaluated, while the collection efficiency will be continuously increased. Manipulations during radiochemical processing will be reduced by fully automating the procedure. Automation of the full radiochemical process will increase time-efficiency, hence leading to less decay of the HSA ^153^Sm, and will enhance process safety.

### Radiolabeling of High Specific Activity ^153^Sm

**Figure 5:**
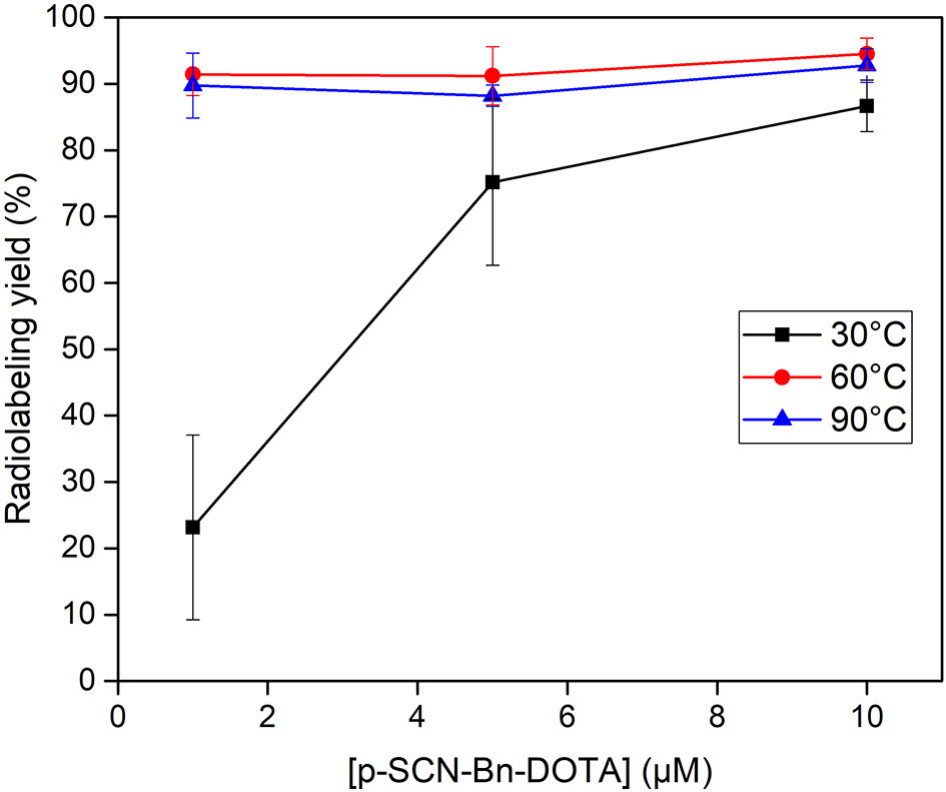
Radiolabeling of ^153^Sm with various concentrations of *p*-SCN-Bn-DOTA at 30 (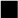), 60 (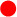) and 90°C (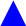) in a 0.1 mol/L NaOAc buffered solution (pH 4.7).

Radiolabeling experiments were performed to investigate applicability of the produced HSA ^153^Sm for TRNT. Radiolabeling was performed in the presence of different concentrations of *p*-SCN-Bn-DOTA. Radiolabeling reactions containing 0.5, 0.1 and 0.05 MBq/nmol were used to evaluate the radiolabeling of the produced HSA ^153^Sm, i.e., having a 5,000, 25,000, and 50,000 stoichiometric excess, respectively, of ligand with respect to ^153^Sm. *p*-SCN-Bn-DOTA was selected as chelator in this study because it is a well-established chelator that is commonly used in TRNT applications with multiple radiolanthanides (33, 34). Radiolabeling was performed at different temperatures and different ligand concentrations (Figure 5). Incubating the reactions at 30 °C was found to be insufficient to form a radiocomplex at low concentrations (radiochemical yield 23.1 ± 13.9% at 1 μmol/L *p*-SCN-Bn-DOTA). This could be expected considering the rigid structure of DOTA which generally requires energy for the radionuclide to be incorporated into the chelator (35). When using high ligand concentrations, higher radiolabeling yields were observed, however the yields were not as high as the yields at elevated temperatures (75.2 ± 12.6% at 30 °C, 5 μmol/L; 86.7 ± 3.9% at 30 °C, 10 μmol/L). Radiolabeling of *p*-SCN-Bn-DOTA at 60 and 90 °C resulted in much higher radiochemical yields in all investigated ligand concentrations. Even when using low concentrations of *p*-SCN-Bn-DOTA, radiolabeling yields were almost quantitative (91.4 ± 3.2% at 60 °C and 89.8 ± 4.9% at 90 °C using 1 μmol/L DOTA). These results clearly demonstrate the applicability of the produced HSA ^153^SmCl_3_. Radiolabeling conditions still require further optimization to achieve maximal molar activity and radiolabeling yields. However, we already demonstrated the ability to achieve radiolabeling yields which are acceptable for radiopharmaceutical production in TRNT. Further optimization and application of ^153^Sm radiolabeled radiopharmaceuticals in preclinical tests are currently ongoing.

## Conclusions

^153^Sm has highly interesting decay characteristics to be used as a theranostic radionuclide. However, its application in TRNT is hampered by its low specific activity as a result of the carrier-added production route for ^153^Sm. In this work we demonstrated the proof-of-concept to produce ^153^Sm with high specific activity by combining neutron activation in a high-flux nuclear research reactor with off-line mass separation. Ionization of samarium for mass separation was enhanced by resonant laser ionization, taking advantage of the exceptional case that only one laser is required for both transitions. The ionization rate could be increased by 30% by doubling the standard repetition rate to 20 kHz. Over the different collections, an average mass separation efficiency of 4.5% could be reached, with a maximum of 12.7%. ^153^Sm could be recovered from the MS collection foil by applying a variety of radiochemical processing steps, obtaining a ^153^SmCl_3_ solution suitable for radiolabeling. The specific activity of 1.87 TBq/mg at the time of MS collection was achieved, i.e., a ^152^Sm-to-^153^Sm ratio of 8:1. Near-quantitative radiolabeling yields of HSA ^153^Sm with various concentrations of *p*-SCN-Bn-DOTA were obtained at 60 and 90 °C, demonstrating the applicability of the produced HSA ^153^SmCl_3_ for radiolabeling.

## Data Availability Statement

The original contributions presented in the study are included in the article/Supplementary Material, further inquiries can be directed to the corresponding authors.

## Author Contributions

BP: neutron activation. MV and AB: radiochemical processing. RH: laser ionization. LL, EC, MVS, and TSc: mass separation. MV, MO, and AB: radiolabeling. MV and CD: project coordination. MV, TEC, MO, TC, TSt, and AB: conceptualization. MV, CD, and RH: initial draft manuscript. All authors contributed to the article and approved the submitted version.

## Conflict of Interest

The authors declare that the research was conducted in the absence of any commercial or financial relationships that could be construed as a potential conflict of interest.
