# Supplementary material for: Production of Sm-153 With Very High Specific Activity for Targeted Radionuclide Therapy
Source: Front Med (Lausanne). 2021 Jul 19;8:675221. doi: 10.3389/fmed.2021.675221 (PMC8326506; doi:10.3389/fmed.2021.675221)
Supplement: Supplementary file 1 [file Data_Sheet_1.PDF]

Supplementary Material

1.1 Chemical purity - ICP-MS data

^{52}Cr (ug/L)	^{55}Mn (ug/L)	^{56}Fe (ug/L)	^{58}Ni (ug/L)	^{59}Co (ug/L)	^{60}Ni (ug/L)	^{63}Cu (ug/L)	^{64}Zn (ug/L)	^{65}Cu (ug/L)	^{66}Zn (ug/L)	^{68}Zn (ug/L)	^{208}Pb (ug/L)
0.32	0.82	16.86	2.17	0.020	2.12	4.68	58.81	4.81	60.40	60.34	126.24

Supplementary Table 1: Chemical purity of the ^{153}Sm fraction after elution from the DGA column. The DGA column loaded with ^{153}Sm was washed with 5 mL of 4 mol/L HNO_3, after which the ^{153}Sm was eluted with H_2O.

^{64}Zn (ug/L)	^{208}Pb (ug/L)
<20	7.1

Supplementary Table 2: Zinc and lead content in the ^{153}Sm fraction after elution from the DGA column. The DGA column loaded with ^{153}Sm was washed with 20 mL of 4 mol/L HNO_3, after which the ^{153}Sm was eluted with H_2O.

2 Validation of the iTLC method

5 µl of the HSA $^{153}SmCl_3$ solution and the radiolabeling solution ($^{153}SmCl_3$ + *p*-SCN-Bn-DOTA) were spotted on separate iTLC papers, and eluted using an acetonitrile/water mixture (70/30) to allow migration of the ^{153}Sm-*p*-SCN-Bn-DOTA complex. After full migration of the front, the iTLC papers were cut in half. The top and bottom part of each iTLC paper was counted separately using a gamma counter (Perking Elmer). When HSA $^{153}SmCl_3$ is eluted with the acetonitrile/water mixture, large majority of the activity is retained at the bottom of the strip. Contrarily, when *p*-SCN-Bn-DOTA is added to the HSA $^{153}SmCl_3$, the resulting ^{153}Sm-*p*-SCN-Bn-DOTA is eluted to the top of the strip.

	Free HSA $^{153}SmCl_3$		$^{153}SmCl_3$ + *p*-SCN-Bn-DOTA	
	CPM	%	CPM	%
Activity top	16177.39	0.7	73967.82	94.5
Activity bottom	2319447	99.3	4304.91	4.5

Supplementary Table 3: Validation of the iTLC method by comparing the migration of ^{153}Sm-*p*-SCN-Bn-DOTA to free HSA $^{153}SmCl_3$.
